# The Dissection Bill

**Published:** 1887-09

**Authors:** 


### THE DISSECTION BILL.

The bill for protecting cemeteries and facilitating the study of practical anatomy has passed the Georgia Legislature and is now a law. The bill as at first proposed contained features not wholly acceptable to the medical colleges, but through the efforts of the editors of this journal and others amendments were secured which in some measure removed the objections, and we are for the first time able to say that we now at least have a color of law for obtaining dissecting material for the all-important study of anatomy.

To Mr. C. M. Candler, representative from DeKalb county, an intelligent and working young member who framed and introduced the bill, and to the kindly influence and aid of physicians among the legislators, together with the assistance of non-professional members of enlarged and progressive views, we are indebted for the passage of this law. And we do not forget to express our appreciation of the good sense of Governor Gordon for his approval of the act.

